# 3D Generation of Multipurpose Atomic Force Microscopy Tips

**DOI:** 10.1002/advs.202201489

**Published:** 2022-07-19

**Authors:** Ayoub Glia, Muhammedin Deliorman, Mohammad A. Qasaimeh

**Affiliations:** ^1^ Division of Engineering New York University Abu Dhabi (NYUAD) Abu Dhabi UAE; ^2^ Department of Mechanical and Aerospace Engineering New York University Brooklyn NY 11201 USA

**Keywords:** 3D printing, carbon nanotubes, focused ion beam, high‐resolution imaging, high‐speed imaging, polymeric atomic force microscopy tips

## Abstract

In this work, 3D polymeric atomic force microscopy (AFM) tips, referred to as 3DTIPs, are manufactured with great flexibility in design and function using two‐photon polymerization. With the technology holding a great potential in developing next‐generation AFM tips, 3DTIPs prove effective in obtaining high‐resolution and high‐speed AFM images in air and liquid environments, using common AFM modes. In particular, it is shown that the 3DTIPs provide high‐resolution imaging due to their extremely low Hamaker constant, high speed scanning rates due to their low quality factor, and high durability due to their soft nature and minimal isotropic tip wear; the three important features for advancing AFM studies. It is also shown that refining the tip end of the 3DTIPs by focused ion beam etching and by carbon nanotube inclusion substantially extends their functionality in high‐resolution AFM imaging, reaching angstrom scales. Altogether, the multifunctional capabilities of 3DTIPs can bring next‐generation AFM tips to routine and advanced AFM applications, and expand the fields of high speed AFM imaging and biological force measurements.

## Graphical abstract

Multipurpose polymeric atomic force microscopy (AFM) tips, named 3DTIPs, are presented with low Hamaker constant for high resolution, high speed AFM imaging. The 3DTIPs are generated with great flexibility in design, material, and function allowing better control over resonance frequency, quality factor, and spring constant. Their tip ends are further post‐processed to extend their functionality in high resolution AFM imaging.

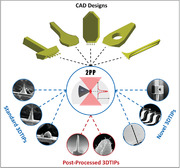

## 1 Introduction

Atomic force microscopy (AFM) has long been recognized for its significance in high resolution imaging in both air and liquid environments. Through rapid evolution over the years reaching angstrom‐scale resolution, AFM is proven to be a key tool to landmark findings such as uncovering crystal structure of inorganic surfaces and spatially resolving molecular interactions within protein complexes.^[^ ^1^, ^2^, ^3^, ^4^ ^]^ Other notable applications of high resolution AFM include time‐lapse imaging of RNA replications by polymerase^[^ ^5^ ^]^ and photo‐actuated myosin V translations on actin filaments^[^ ^6^ ^]^ at high speed scan rates. However, while the principal features of AFM tips used in these studies, and in general, are silicon‐based 3D structures, their fabrication is tedious and geometrically restricted to 2D micromachining.^[^ ^7^ ^]^ Moreover, silicon tips are brittle with susceptibility to wear and contamination, which substantially degrade the overall imaging resolution and their reliability when scanning is performed over extended durations.^[^ ^8^ ^]^ Therefore, there is a continuous demand for alternative materials, simpler microfabrication approaches, and innovative concepts to serve for next‐generation AFM tips. An ideal AFM tip would offer extended degree of freedom to designs, multifunctionality to work in air and liquid with high resolution and speed, reduced tip wear and contamination during imaging, and the capability to probe soft matter with high precision.

Two‐photon polymerization (2PP) technique is rapidly revolutionizing 3D microfabrication due to flexibility at which it facilitates obtaining structures that are otherwise “deemed impractical” by standard 2D micromachining.^[^ ^9^ ^]^ Indeed, 2PP is a powerful technology that prints, layer by layer, 3D structures at the micro/nanoscale with a resolution that extends to the sub diffraction limit.^[^ ^10^ ^]^ In the field of AFM, 2PP offers the capability of seamlessly integrating complex‐shaped tips and cantilevers in a single polymer‐additive manufacturing process. However, since the properties of polymeric resins significantly vary from that of silicon, their use in microfabricating AFM tips requires detailed characterization and optimization to ensure fitness for already established tip characteristic standards. This way, with the use of alternative materials, the AFM imaging limitations related to the spatial resolution, scanning speed, and durability during long scans could be foremost addressed and overcome.

In recent years, several different approaches were considered to microfabricate AFM tips with the aim to overcome some of the above‐mentioned limitations.^[^ ^11^, ^12^, ^13^, ^14^ ^]^ Among them, utilizing 2PP in the fabrication of AFM probes was mainly due to its true 3D control over the resulting tip's geometry and aspect ratio. In the studies, it has been shown that the hydrophobic polymeric AFM tips produced by 2PP minimize adhesion forces between the tip and sample during probing.^[^ ^14^, ^15^ ^]^ In addition, it has also been shown that the intrinsic properties of resins permit larger frequency bandwidths,^[^ ^9^ ^]^ which is relevant for high speed imaging.^[^ ^16^ ^]^ Together, all these are valuable works for investigating the 3D printing effect on the resulting probe properties such as the density, stiffness, and the fundamental resonance frequency. However, there is still a lack of understanding of the material‐based differences between silicon and epoxy AFM tips in terms of tip wear, their contribution to the forces of interaction, and temporal resolution limits (i.e., tip response time). Moreover, equally important is to reproducibly fabricate the tips and thoroughly investigate their functionality in all AFM operating modes in order to effectively probe a myriad of samples in different environmental settings.

In this work, 2PP is deployed to generate multipurpose epoxy resin (SU8)‐based AFM tips (referred to as 3DTIPs) with great flexibility in design and function. Parametric analysis is first carried to benchmark the frequency domain of the 3DTIPs against their silicon counterparts in terms of resonance frequency, oscillation modes, and spring constant. Subsequently, 3DTIPs are experimentally benchmarked for image quality, tip wear, durability over extended scanning durations, high resolution, and scanning speeds. In doing so, common AFM modes (contact, dynamic, and PeakForce) were employed in liquid and air to investigate the imaging capacity on different samples such as polystyrene (PS) nanospheres, gold grains, plasmid DNA, and antibodies. Two additional advancement of 3DTIPs, one refined by using focused ion beam (FIB) process and one integrated with far‐reaching carbon nanotubes (CNTs), are also characterized and compared for their high aspect ratio (HAR) functionality by performing a series of high resolution imaging on plasmid DNA. Finally, CNT‐integrated 3DTIPs are demonstrated to resolve the atomic resolution of highly ordered pyrolytic graphite (HOPG) sample. All of which makes the technology a potential candidate for revolutionizing the field by introducing the 3D generation of multipurpose AFM tips.

## 2 Results

### 2.1 3DTIP Concept

**Figure 1:**
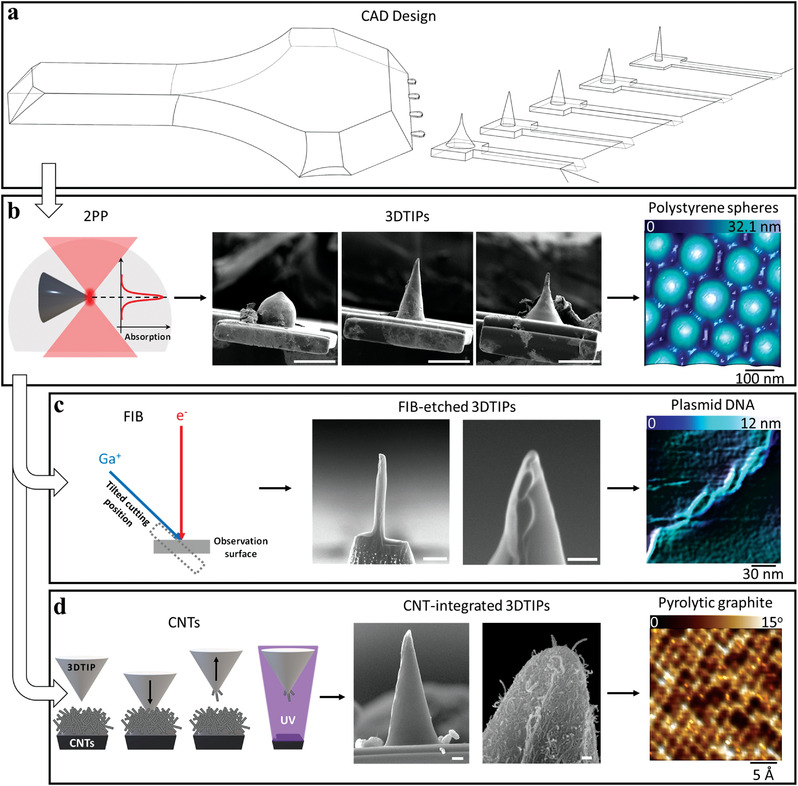
3D generation of multipurpose AFM tips (3DTIPs). a) CAD design of a 3DTIP showing the mounting base and the array of cantilever‐mounted tips with different geometries. b) Left panel: Schematic showing 2PP working principle for single‐step microfabrication of the 3DTIPs via polymer‐additive manufacturing process. Middle panel, left to right: SEM images reveal that with 2PP, generation of multipurpose 3DTIPs (bead, conical, and HAR) is possible. Scale bars: 25 µm. Right panel: AFM height sensor image, obtained by scanning, in contact mode in air, PS spheres with a conical 3DTIP, reveals high resolution imaging capability. c) Left panel: Schematic showing FIB working principle for etching the tip end of the 3DTIPs. Middle panel: SEM images showing a 3DTIP with enhanced aspect ratio and reduced tip radius. Scale bars: 2 µm and 100 nm (left to right). Right panel: AFM height sensor image, obtained by dynamic scanning plasmid DNA with FIB‐etched 3DTIPs in liquid, reveals enhancement in resolving fine 3D nanostructures. d) Left panel: Schematic showing the steps involved in incorporating the tip end of a 3DTIP with randomly oriented CNTs. Middle panel: SEM images showing the far‐reaching CNTs at the tip end of the CNT‐integrated 3DTIPs. Scale bars: 4 µm and 200 nm (left to right). Right panel: AFM height sensor image, obtained by scanning, in dynamic mode in liquid, pyrolytic graphite with CNT‐integrated 3DTIP in air, reveals the true atomic resolution of its highly ordered structures.

The main concept behind 3DTIPs is the advancement of AFM tips with innovative 3D designs, thus extending their capabilities and functionalities. To date, majority of AFM tips are developed based on silicon‐based subtractive micromachining, which imposes limitations to their design and materials used. Here, on the other hand, we explore the potential for using single polymer‐additive manufacturing to achieve true 3D AFM tips for wide‐range of different applications. Hence, our work puts forward the 2PP strategy for fabrication of 3DTIPs and explores their use in the area of high resolution, high speed AFM imaging. The main 3DTIP fabrication workflow constitutes of 3D CAD design and 3D printing (**Figure** **1**a,b). Our fabrication workflow also introduces two additional post‐fabrication processing steps with the aim to further advance 3DTIP performance. In particular, FIB etching and CNT inclusion are explored for this purpose and are used to increase the tip aspect ratio and reduce its apex end size (Figure 1c,d).

First, the 3DTIPs are designed with inbuilt components (mounting base, cantilever, and tip) to ensure consistency across probe properties (Figure 1a; Figure S1a, Supporting Information). As such, with careful consideration, the base can take any geometry and shape suitable for mounting the 3DTIPs on any commercial AFM tip holders. Importantly, in each 3DTIP, cantilevers and tips can comprise identical geometries and shapes. This way, the arrays of 3DTIPs, having constant separation distance between cantilevers, can be used independently or in parallel for imaging and applications involving surface modifications (e.g., dip pen nanolithography).^[^ ^17^ ^]^ Second, the 3DTIP fabrication is performed in one step via 3D printing using SU8 resin and a commercial 2PP system (Figure 1b). The high flexibility of SU8 and high resolution of 2PP enable rapid manufacturing of different shaped cantilevers (e.g., rectangular, triangular) and tips (e.g., bead, conical, and HAR) suitable for common force measurements and high resolution imaging. Noteworthy, with proper designs, the printing time of the 3DTIPs comprising the base and array of cantilever‐mounted tips can be as low as 15 min (Figure S1, Supporting Information) to as high as ≈1.5 h (this study). To reduce the printing time even further and make production of the 3DTIPs more suitable for high volume manufacturing, the cantilevers and the tips can be printed on previously manufactured SU8‐based mounting bases, which alone constitutes about 99.9996% of the total printed volume. The production time of 3DTIPs could then be as low as 1 3DTIP per minute.

Third, the 3DTIPs can be further nanomachined with FIB technology to extend their aspect ratio beyond the resolution of 2PP (Figure 1c). With this, higher resolution is provided for imaging applications involving 3D nanostructures. Fourth, the 3DTIPs can also be integrated with CNTs as another post‐processing procedure (Figure 1d). Thanks to the plausible physical properties of CNTs (e.g., sharp tip, HAR, and being very strong along their axial direction),^[^ ^18^ ^]^ with the CNT‐integrated 3DTIPs resolving highly ordered structures with atomic resolution is achievable.

### 2.2 3DTIP Characterization

**Figure 2:**
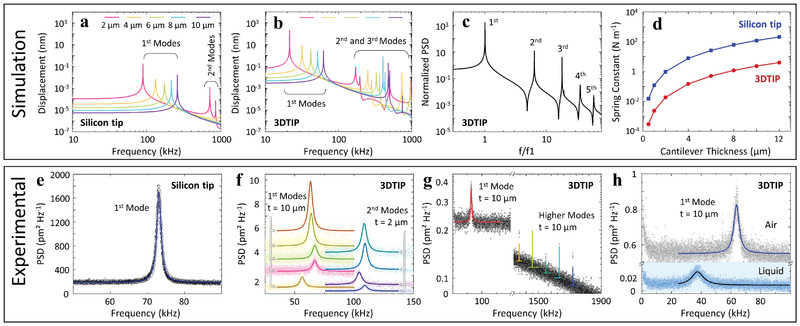
Parametric benchmarking of the 3DTIPs against silicon cantilevers. a) Simulation results for a range of silicon cantilever thicknesses (denoted as *t*) reveal their first and second oscillation modes. b) In comparison, the simulation results for the 3DTIPs with identical cantilever thicknesses reveal wider range of frequency domain. c) Power spectral density (PSD) shows several oscillation modes for the 3DTIPs, which could be suitable for various AFM measurements. d) For varying cantilever thicknesses, the spring constants of the 3DTIPs are an order of magnitude lower compared to silicon tips, suggesting their use for sensitive force measurements. e) A typical first oscillation mode of a standard silicon tip (*t =* 0.5 µm) in air. Solid blue line: Lorentzian fit to data. f) The PSD shows the first and second oscillation modes of the 3DTIPs in air with *t =* 10 µm and *t =* 2 µm cantilever thicknesses, respectively. g) With the 3DTIPs, obtaining multiple higher oscillation modes in air that span wide tuning range (up to 2 MHz) is possible. h) The PSD shows the first oscillation modes of the 3DTIP with *t =* 10 µm cantilever thickness in air and liquid. Solid blue line: Lorentzian fit to data.

When fabricating AFM tips, it is desired that, in addition to their fundamental resonance frequency, higher oscillation modes can be detected. As such, investigating the frequency domain of the 3DTIPs for higher oscillation modes is important to correlate the cantilever dimensions and material properties to the overall probe characteristics, such as fundamental frequency, quality factor, deflection sensitivity, and stiffness. Therefore, taking into consideration that the frequency response is highly dependent on the tip's material property (e.g., density and Young's modulus of elasticity) and the cantilever dimensions (e.g., length, thickness, or aspect ratio),^[^ ^19^ ^]^ we first benchmarked the frequency domains of 3DTIPs against common conventional silicon tips both by simulation and experiments (**Figure** **2**).

Simulation benchmarking was carried out using comparative analysis of similar tip structures and cantilever dimensions (see Experimental Section). The key parameters such as resonance frequency, *f*, oscillation mode, *w*, and spring constant, *k*, were then mapped using finite element modelling for cantilever thicknesses ranging from 0.5 to 12 µm, while keeping other dimensions identical (Figure 2a–d). Results revealed an order of magnitude lower resonance frequencies for 3DTIP cantilevers compared to their silicon counterparts (Figure 2a,b). This was due to the significantly lower density, *ρ*, and elasticity, *E*, of SU8,^[^ ^20^ ^]^ which is expected according to the dependency of resonance frequency to the material properties as *f* ∝ (*E/ρ*)^1/2^. As a result, extending the measurable oscillation modes of the cantilever to higher frequency ranges (up to 2 MHz), while retaining the cantilever shape and dimensions, is possible (Figure 2c).

Moreover, for given cantilever thicknesses, the 3DTIP spring constants were two orders of magnitude lower than those of silicon (Figure 2d), suggesting that the small changes in the 3DTIP deformation could be measured more precisely during AFM experiments that require higher sensitivity, such as probing soft matter and cell deformation measurements.^[^ ^21^ ^]^ Additional simulations further confirmed similar results for the 3DTIP and silicon cantilevers with varying length, thickness, and aspect ratio (Figure S2, Supporting Information). Together, our analysis revealed that the 3DTIPs, offering wide range of frequency domain, are very good candidates for AFM measurements that require higher oscillation modes in obtaining more precise information on sample properties.

To test the validity of our parametric analysis, next we performed an experimental benchmarking by characterizing the 3DTIPs against a standard silicon tip (SCM‐PIT‐V2, Bruker). The experiments were conducted in air using a commercial AFM (Dimension Icon, Bruker). In the 3DTIPs, the cantilever thicknesses (denoted as *t*) were selected to cover both stiff (*t* = 10 µm) and soft (*t* = 2 µm) probes. Whereas, according to manufacturer, the thickness of the silicon cantilever was *t* = 0.5 µm. In the experiments, the thermal vibration of cantilevers was used to reveal the oscillation modes, and the corresponding quality factor, *Q*, was estimated from Lorentzian fitting of the modes.^[^ ^22^ ^]^

For the silicon cantilever, the single observable frequency of 73 kHz (*Q* = 150) fell within the normal range of the standard silicon tips having similar geometry and dimensions (Figure 2e). While for the 3DTIP cantilevers, the thermal tuning measurements presented a very good level of consistency with simulations (Figure 2f). For example, the first frequency of 62.3 kHz ± 5 kHz (*Q* = 9) with *t* = 10 µm and the second frequency of 105.6 kHz ± 3 kHz (*Q* = 15.5) with *t* = 2 µm were within the range of their corresponding predicted values, 60.5 and 106.7 kHz, respectively (Figure 2b). Furthermore, for the 3DTIP cantilevers, higher oscillation modes spanning over a 2 MHz spectrum were also observed (Figure 2g). This was expected as the lower density and elasticity of SU8 caused a shift in resonance frequencies, which made more oscillation modes to appear in the spectrum and produced a lower quality factor, an indication of energy dissipation over shorter time periods. This result further substantiated the validity of our parametric analysis and reinforced their wider range applicability for when imaging in liquid environment. For example, lower quality factor further permits higher dynamic mode scanning rates that are suitable for real‐time capture of high speed biological activities such as the motion of membrane proteins.^[^ ^23^ ^]^ Thus, for applications involving imaging in liquid, first resonance frequency of the 3DTIPs (*t* = 10 µm) was also measured in water (Figure 2h) with a value of 39.42 kHz ± 2 kHz (*Q* = 2.7).

### 2.3 Quality Factor Consideration

Next, we sought to investigate to what extent *Q* factors of 3DTIPs can be increased by decreasing the cantilever length, and elaborate on other design strategies for obtaining higher *Q* factors. The *Q* factor of an AFM tip bears information about the energy dissipation in a cantilever. As such, higher *Q* factors are associated with lower energy dissipations per cycle, given that the stored energy is constant. Therefore, high *Q* factors provide better frequency resolution, and thus are essential for achieving high force sensitivity.

When cantilever is resonating in air, the *Q* factor is mainly attributed to combined dissipation mechanisms of extrinsic and intrinsic nature:^[^ ^24^ ^]^ In the former, the dissipation stems from interactions with the surrounding air, while in the latter, from interactions within the cantilever structure or with its support structure. As a result, for a cantilever with thickness *H* and length *L*, where *f* ∝ *H*/*L* ^2^,^[^ ^24^ ^]^ the *Q* factor is a combination of *Q* factors produced by each dissipation mechanism. Here, dominant energy losses are due to viscosity of the air (with $Q_{\mathrm{Air}}\propto \;\sqrt{H^{3}}/L$) and damping of the support (with *Q*_Clamp_ ∝ *L* ^3^/*H* ^3^), respectively.^[^ ^24^ ^]^

Experimentally, we considered four cantilever designs with constant *H* and linearly decreasing *L* (Type A cantilever, Figure S3a, Supporting Information, top panel), decreasing *H* and *L* that provide constant resonance frequency (Type B cantilever, Figure S3a, Supporting Information, middle panel), and constant *H* and linearly decreasing *L*, but this time of a novel design that comprises two arms extending triangularly and conversing to hold a disk (Type C cantilever, Figure S3a, Supporting Information, bottom panel). In the last consideration, the aim was to investigate two vibration modes: one of the disk and one of the whole cantilever. The *H* and *L* values of the cantilevers are given in Table S1, Supporting Information. Following, thermal vibration of cantilevers were used to reveal the respective oscillation modes, and consequently estimate the corresponding *Q* factors from Lorentzian fitting of the modes.^[^ ^22^ ^]^ Results revealed that in all considerations, the Lorentzian fit showed improved root‐mean‐square deviations as qualitative indication of an increased *Q* factor (Figure S3b–e, Supporting Information). In particular, we obtained that the maximum *Q* factor could be ≈40 (Type C cantilever) which corresponded to an order of magnitude increment (Figure S3f, Supporting Information).

Overall, it was evident that the *Q* factor of 3DTIPs could be increased in air by decreasing cantilever length or changing its design. However, compared to *Q* factor of a typical silicon cantilever in air (≈100–150), the increment in *Q* factor of the 3DTIPs was not substantial. Thus, more systematic research on all *Q* factor dependencies needs to be conducted, which we left for future work. For example, in addition to cantilever size and shape, the *Q* factor also depends on the density, the elastic modulus, and the thermal conductivity of cantilever material. Density and elastic modulus of epoxy‐based resins (e.g., SU8) can be increased by fourfold by increasing the curing temperature, which would result in a significant reduction of the viscous damping in air, and thus 16 times increase of *Q*_Air_. The cantilever can also be coated with a relatively thicker layer of gold, which would result in increased density, elastic modulus, and thermal conductivity, and decreased linear thermal expansion, all of which would contribute to significant increase in the *Q* factor of 3DTIPs.

### 2.4 Image Quality and Tip Wear

**Figure 3:**
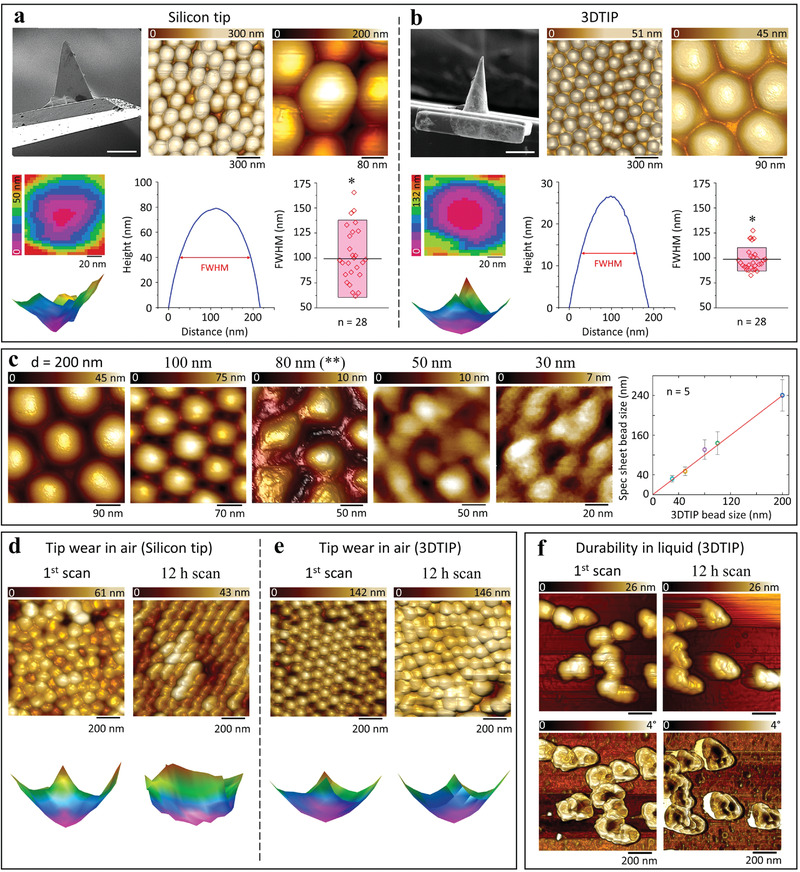
Imaging quality and tip wear of silicon tips and 3DTIPs in air, as well as 3DTIP durability test in liquid. Top left panels: SEM images of a) standard (pyramidal) silicon tip and b) conical 3DTIP used in characterizing PS spheres (scale bars: 25 µm). Top middle and right panels: AFM height sensor images of 200 nm PS spheres obtained at different scales in air using contact mode. Bottom left panels: Bottom and side views of reconstructed (by deconvolution) tip shapes. Bottom middle and right panels: Full width at half maximum (FWHM) of vertical profiles as a function of size of PS spheres. Box plots represent mean ± SD (*n =* 28). ^*^: Significantly different at *p* < 0.05 using two‐sided student's *t*‐test. c) Left to right: AFM height sensor images of PS spheres with sizes *d* = 200, 100, 80, 50, and 30 nm obtained with the 3DTIP in air using contact mode. Here, the change in scales of the images is to reveal the deterioration of the resolution with reduced bead sizes. ^**^: The image acquisition was performed in dynamic mode at 86.6 Hz scan rate (1 frame s^–1^) with 96 × 96 pixel size and 263 × 263 nm^2^ scanning area. The measured sizes of PS spheres also showed good linear correlation (*r* ^2^ > 99) with their manufacturer sizes. Error bars represent mean ± SD (*n =* 5). d,e) Comparison of AFM height sensor images obtained after first scan and after 12 h continuous scanning of 100 nm PS spheres in air using contact mode revealed that the 3DTIPs are more resistant to tip wear than the silicon tips, also verified by the side views of their reconstructed tip shape images. Notably, compared to the shape of the silicon tip, the shape of the 3DTIP shows excellent tip wear resistance following the imaging. f) AFM amplitude (top) and phase (bottom) images of 200 nm PS spheres obtained with the 3DTIP in liquid using dynamic mode (scan rate = 48.8 Hz) revealed that the 3DTIPs are durable in liquid for prolonged scanning durations.

With the experimental results demonstrating that the 3DTIPs offer lower quality factor over wide range of frequency domain, next we explored their AFM imaging quality and tip wear resistance and compared the results with standard silicon tips of similar design (**Figure** **3**).

Typically, AFM imaging of a sample surface is performed either in contact mode, where the tip is in constant physical contact with the sample, or in dynamic mode, where the tip is oscillating over the sample at its resonance frequency. The dominant interaction forces involved in each of the two modes are lateral and vertical, respectively. Hence, the choice of which mode to use widely depends on the application, since each mode has its own practical implications on the image quality (resolution), tip wear, and sample deformation.^[^ ^21^ ^]^ PeakForce, the additional mode we use in this work, has more control over the vertical forces (range of tens of pN), whereby the image is obtained through oscillations of the tip far below its resonance frequency.^[^ ^25^ ^]^ In this “off‐resonance” tapping mode, the maximum interaction force (i.e., peak force) constitutes the main signal for each pixel in the acquired image.^[^ ^26^ ^]^

To test the functionality of the 3DTIPs, PS spheres (200 nm diameter) were immobilized on a glass substrate and AFM imaging was performed in air using contact mode. The image quality and tip wear were then investigated from the height sensor images, since these images contain equal amounts of information on the sample topography and tip shape.^[^ ^27^ ^]^ For benchmarking, a pyramidal silicon tip with *f* = 73 kHz, *t* = 0.5 µm, *k* = 5.6 N m^−1^, *Q* = 150 and a conical 3DTIP with *f* = 62 kHz, *t* = 10 µm, *k* = 0.7 N m^–1^, *Q* = 9 were used (Figure 3a,b). The radius of curvature, *r*, for the 3DTIP was *r* = 150 nm, while for the silicon tip, it was *r* = 20 nm (manufacturer value).

As shown in Figure 3a, the silicon tip was able to reveal the size of PS spheres, but with slightly distorted ellipsoidal profiles. The misrepresentation of PS spheres is attributed to the coarse, irregular, and deformed silicon tip shape (see reconstructed tip shape image in Figure 3a). This shape results from the anisotropic fracturing of silicon tip.^[^ ^28^ ^]^ As such, silicon is brittle by nature, with a crystal structure that makes the tip susceptible to breakage along its favored crystal planes during AFM scans. The 3DTIP, on the other hand, not only revealed the size but also outperformed the silicon tip by visually revealing spherical shape of PS spheres (Figure 3b). This was attributed to the flexible molecular structure of the SU8,^[^ ^20^ ^]^ which substantially caused the tip shape of the 3DTIP to experience isotropic wear during imaging (see reconstructed tip shape image in Figure 3b). For both tips, FWHM of vertical profiles as a function of PS size is also shown in Figure 3a,b. Compared to silicon tip (mean FWHM value of 99.1 ± 38.5 nm), 3DTIP provided significantly narrower FWHM in the shape with mean value of 98.5 ± 11.7 nm. Additional AFM imaging on PS spheres further confirmed that the 3DTIPs are suitable for obtaining high quality images in a reproducible manner (Figure S4, Supporting Information).

The fact that 3DTIPs achieve similar, and in some cases, higher image resolutions than silicon tips (even when the tip radius in the former is ≈7.5 times larger) can be explained by their milder surface roughness and higher aspect ratio. The latter, in particular, is known to be a key parameter in providing full vertical access to 3D nanostructures.^[^ ^29^ ^]^ These attributes aside, the Hamaker constant of SU8, the magnitude of which reflects the strength of interaction between the tip and sample,^[^ ^30^ ^]^ makes it more important contribution to the image quality due to significantly lower and extremely localized forces of interaction between the 3DTIP and sample surface. Although, the short‐range forces between the tip and sample are mainly attributed to van der Waals interactions, which result from fluctuations in the electrical dipole of atoms^[^ ^7^, ^31^ ^]^ and are limited to interacting particle sizes of a few hundred angstroms,^[^ ^26^, ^32^ ^]^ they are generalized into macroscopic bodies using the Hamaker approach^[^ ^30^ ^]^ and their potential is calculated using Israelachvili.^[^ ^31^ ^]^

The relation between the above two approaches is given by *F*_VdW_ = *RH*/(6*d* ^2^), where *F*_VdW_ is the van der Waals force of interaction, *R* is the tip radius, *H* is the Hamaker constant, and *d* is the tip‐sample separation distance.^[^ ^30^ ^]^ With *H* = *π* ^2^ *q*_1_ *q*_2_ *λ*_12_ describing two‐body (tip‐sample) interaction,^[^ ^28^ ^]^ where *q*_1_ and *q*_2_ are the number densities of the interacting bodies and *λ*_12_ is their interaction coefficient which involves polarizabilities and characteristic atom potentials; and with $H\;=\pi^{2}\;\left(q_{1}q_{2}\lambda_{12}+q_{0}^{2}\lambda_{00}-q_{0}q_{1}\lambda_{01}-q_{0}q_{2}\lambda_{02}\right)$ describing three‐body (tip‐sample interaction in liquid as denoted by suffix 0), Hamaker constants can be theoretically calculated. For silicon nitride–silicon nitride interaction, Hamaker constants are reported to be 17.3 × 10^–20^ J in air and 5.3 × 10^–20^ J in water.^[^ ^33^ ^]^ While for SU8‐silicon interaction, these values are reported to be 0.65 × 10^–20^ J in air and 0.18 × 10^–20^ J in water.^[^ ^34^ ^]^ Thus, with the latter Hamaker being ≈26 times smaller in air and ≈30 times smaller in water, the magnitude of the van der Waals interactions is also significantly less with the 3DTIPs, which makes them prone to screening and overshadowing by other forces rising from the tip‐sample environment, such as air, liquid, and humidity.

The influence of Hamaker constant on minimizing the overall forces of interaction between the 3DTIP and the substrate (and thus enhancing imaging resolution) is further emphasized when a gold‐coatd and glass substrate was imaged in air using dynamic mode (Figure S5, Supporting Information). As such, with the 3DTIP having tip radius of *r* = 150 nm, gold grains with 20 nm diameter size were successfully visualized in air even when the imaging is performed at relatively high scan rates (48.8 Hz).

Next, PS spheres with diameters of *d* = 200, 100, 80, 50, and 30 nm were used to examine the imaging performance of the 3DTIPs in air through recovering the exact size and shape of the spheres. As can be seen in Figure 3c, the height sensor images revealed the actual size and shape of all tested PS spheres, with a slight distortion of the shape in case of 30 and 50 nm spheres. Notably, the height sensor image (96 × 96 pixels and 263 × 263 nm^2^ scanning area) obtained with 80 nm PS spheres was acquired at a 1 frame s^–1^ scan rate (86.6 Hz) in dynamic mode, a promising temporal resolution that could be beneficial for high speed AFM imaging.

Because tip wear becomes increasingly prevalent when imaging duration increases from minutes to hours, the resilience of the 3DTIPs for long term scanning was also benchmarked against silicon tips by continuously imaging (for a duration of 12 h) 100 nm PS spheres. Here, the experiments were conducted in air using PeakForce mode. With the silicon tips, resulting height sensor and tip reconstruction images clearly suggested increased deterioration in the image quality, and hence increased anisotropic tip wear (Figure 3d). While with the 3DTIPs, preserved imaging resolution suggested significant tip resilience due to isotropic tip wear (Figure 3e). Furthermore, continuous acquisition of amplitude and phase images of the PS spheres in liquid using dynamic mode (scan rate = 48.8 Hz) clearly distinguished the spheres after 12 h of scanning, with sizes and shapes comparable to the first scan (Figure 3f). This result reinforced the relatively high imaging and phase contrast resolutions of the 3DTIPs, hence suggesting a high tip wear resistance when compared to silicon counterparts.

### 2.5 High Resolution Imaging

**Figure 4:**
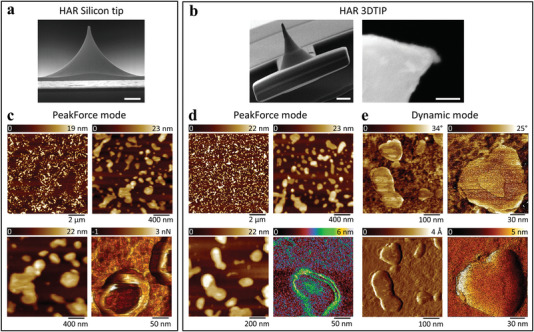
Imaging of plasmid DNA in liquid with HAR silicon tip and HAR 3DTIP using PeakForce and dynamic mode. SEM images of a) the HAR silicon tip (scale bar: 2 µm) and b) the HAR 3DTIP (scale bar: 10 µm) used in imaging plasmid DNA. The zoomed image in (b) shows the tip shape and size of the 3DTIP (scale bar: 100 nm). c,d) AFM height sensor (top two and bottom left), and bottom right c) adhesion and d) deformation images of plasmid DNA obtained with HAR silicon tip (c) and HAR 3DTIP (d) in liquid using PeakForce mode (scan rate = 24.4 Hz). The height images revealed ≈30 nm apparent thickness of the plasmid DNA. e) AFM phase (top two) and amplitude (bottom two) images of plasmid DNA obtained with HAR 3DTIP in liquid using dynamic mode (scan rate = 24.4 Hz). In phase images, a clear distinction between the DNA and mica is also observed.

After characterizing the 3DTIPs for image quality and tip wear, demonstrating their improved durability for imaging in liquid, and exploring their resolution limits by imaging 30 nm PS spheres, we next focused on high resolution imaging of soft biological samples including plasmid DNA (**Figure** **4** **;** Figure S6, Supporting Information) and antibodies of epithelial origin (Figure S7, Supporting Information). As before, the functionality of the 3DTIPs was benchmarked against the silicon tips. This time, however, for resolving the fine nanometer‐scale structures of the samples, a HAR silicon tip (NT Biotool v0020‐5, NanoAndMore) with a tip radius of 2 nm (Figure 4a) and a HAR 3DTIP with a tip radius of 30 nm (Figure 4b) were used. The scan rate in the experiments was set to 24.4 Hz.

The 2PP technology brings complete control over tip height/base aspect ratio at one printing step, which in turn allows for seamless 3D generation of smooth HAR 3DTIPs (Figure 4b). Indeed, compared to silicon tip sharpening approaches, which involve chemical processes such as dry or wet isotropic etching followed by thermal oxidation,^[^ ^35^ ^]^ the technology of printing HAR 3DTIPs relies on optimizing 2PP key running parameters, such as hatching and slicing distances, scanning speed, and laser power (see Experimental Section). Moreover, in the silicon tip sharpening, the tip roughness is reduced by increasing oxidation temperature.^[^ ^35^ ^]^ While in the 2PP, the HAR 3DTIP roughness is reduced by maximum overlapping hatching and slicing distances.^[^ ^16^ ^]^

Moving forward, following immobilization of DNA on mica, the AFM imaging was performed in liquid using PeakForce mode. For both tips, height sensor, adhesion, and deformation images clearly revealed significant decrease in background noise levels due to imaging in liquid (Figure 4c,d). However, the observable (apparent) DNA thickness in the images appeared to be ≈30 nm. This overestimation in DNA thickness was attributed to the relatively larger tip radii of both tips in comparison to the true DNA thickness (≈2 nm).^[^ ^36^ ^]^ Nevertheless, for the imaging of antibodies, height sensor images revealed their presence on the glass substrate, while height sensor and amplitude images distinctively revealed their height (≈2 to 6 nm) (Figure S7, Supporting Information). This observation was in agreement with previously reported size of antibodies using high resolution AFM imaging.^[^ ^37^ ^]^

The HAR 3DTIP was also used to perform dynamic mode scans on the plasmid DNA in liquid. Phase images revealed a clear distinction between the DNA and mica (Figure 4e). However, the ability to perform high speed scans while reconstructing a more realistic DNA strand thickness required the examination of the temporal resolution limits of the 3DTIPs and further modification of their tip ends, both of which are addressed in the next two sections.

### 2.6 High Speed Imaging Capability

**Figure 5:**
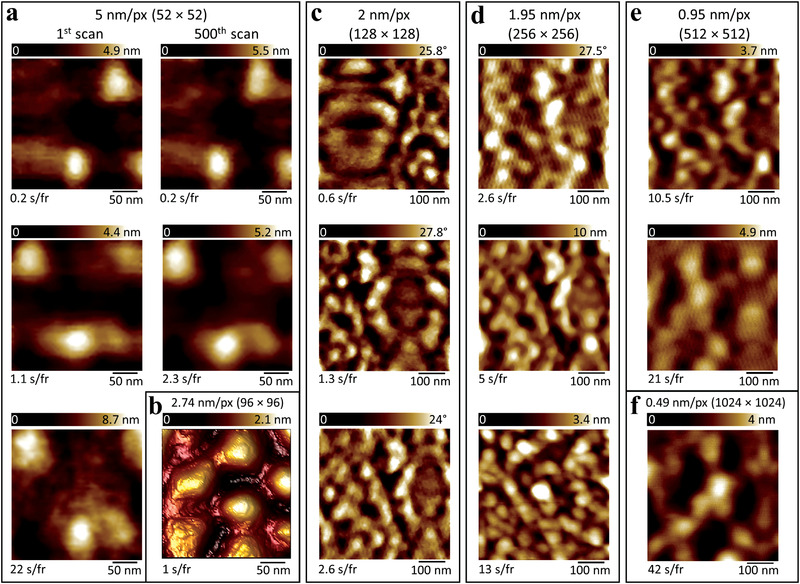
High speed imaging performance of conical HAR 3DTIPs (*r =* 30 nm) in liquid using dynamic mode. a) AFM height sensor images of 50 nm PS spheres obtained by scanning over 263 × 263 nm^2^ area at 0.2, 1.1, 2.3, and 22 s per frame scan rates, which corresponded to 67.6, 12.3, 5.9, and 0.6 µm s^–1^ scan speeds, respectively. No visible difference in the shape of PS spheres was observed between the first and 500th scan at 67.6 µm s^–1^ scan speed. Whereas increasing the scan speed from 0.6 to 12.3 µm s^–1^ resulted in slight decrease in the quality of images at each pixel. b) AFM height sensor image of 80 nm PS spheres obtained by scanning over 263 × 263 nm^2^ area at 1 s per frame scan rate, which corresponded to 25.2 µm s^–1^ scan speed. c) AFM phase images of 30 nm PS spheres obtained by scanning over 500 × 500 nm^2^ at 0.6, 1.3, and 2.6 s per frame scan rates, which corresponded to 54.6, 25.2, and 12.6 µm s^–1^ scan speeds, respectively. d) AFM phase (top) and height sensor (middle and bottom) images of 30 nm PS spheres obtained by scanning over 500 × 500 nm^2^ area at 2.6, 5, and 13 s per frame scan rates, which corresponded to 49.1, 25.6, and 9.8 µm s^–1^ scan speeds, respectively. e) AFM height sensor images of 30 nm (top) and 50 nm (middle) PS spheres obtained by scanning over 500 × 500 nm^2^ area at 10.5 and 21 s per frame scan rates, which corresponded to 23.7 and 11.9 µm s^–1^ scan speeds, respectively. f) AFM height sensor image of 50 nm PS spheres obtained by scanning over 500 × 500 nm^2^ area at 42 s per frame scan rate, which corresponded to 12.2 µm s^–1^ scan speed.

Next, we sought to investigate the high speed capability of the 3DTIPs. For this, a conical HAR 3DTIP (*r* = 30 nm) was tested by performing AFM scans on 50 nm PS spheres in liquid using dynamic mode. A range of number of pixels (from 52 × 52 to 1024 × 1024) were then considered for each frame comprising scanning areas of either 263 × 263 or 500 × 500 nm^2^. This provided pixel size of 5, 2.74, 2, 1.95, 0.95, and 0.49 nm (**Figure** **5**). Additionally, scan times ranging from 42 to 0.2 s per frame were applied to investigate the tip response time for each image. This provided scan speeds ranging from 5.9 to 67.6 µm s^–1^ (Figure 5).

With the 3DTIP, the spatial resolution in a 52 × 52 pixels image (263 × 263 nm^2^ scan area; 5 nm pixel size) was sufficient enough to reveal the size and shape of PS spheres at 0.2 s per frame scan rate, which corresponded to 67.6 µm s^–1^ scan speed (Figure 5a, top left panel). With the silicon tip (similar design), on the other hand, it took more than 30 s per frame to retain the PS shape and size. As expected, continuous scanning of the same area for 500 times with the 3DTIP did not visibly affect the spatial resolution (Figure 5a, top right panel). Additional imaging using scan rates of 22, 2.3, and 1.1 s per frame revealed a slight decrease in the image quality with increasing scan speeds of 0.6, 5.9, and 12.3 µm s^–1^, respectively (Figure 5a). However, in the images, the overall shape and size of PS spheres were not substantially compromised.

Following, our intention was to inspect the extent to which high speed scanning can be applied (i.e., pixel number and scan area limits) while visibly maintaining the image of 50 nm PS spheres. For this, imaging 96 × 96, 128 × 128, 256 × 256, 512 × 512, and 1024 × 1024 pixels (500 × 500 nm^2^ scan area; 2.74, 2, 1.95, 0.95, and 0.49 nm pixel size, respectively) were considered. Results in Figure 5b–f reveal that effective temporal resolutions ranging from 42 to 0.6 s per frame could be provided, with corresponding scan speeds ranging from 12.2 to 54.6 to µm s^–1^.

The ability of the 3DTIPs to perform high speed imaging can be explained by the tip response time, *τ*, which is proportional to the ratio of quality factor, *Q*, and inversly proportional to the fundamental resonance frequency, *f*_1_ (i.e., *τ* = *Q*/*πf*_1_). Accordingly, the short tip response time is achieved by minimizing *Q*/*f*_1_. Thus, with their significantly lower *Q*, the 3DTIPs are more suitable for high‐speed imaging compared to silicon tips having similar *f*_1_. For example, a 3DTIP with *f*_1_ ranging from 40 to 100 kHz has *Q* ranging from 2 to 10 in liquid, which could result in a tip response time of 10 to 250 µs per pixel. These tip response times turn to be 10 to 40 times shorter than the response times of a standard silicon tip. However, it is also important to note that in a typical AFM system, the maximum imaging rate is also limited by various factors, including speed performance of the feedback bandwidth, scan size, number of scan lines, spatial frequency of feature height corrugation, and as well, phase delays in the feedback operation, which are affected by the sample fragility.^[^ ^38^ ^]^ Therefore, achieving higher scanning speeds^[^ ^39^ ^]^ than reported in this study will require careful consideration of theabove‐mentioned AFM‐related factors when improving the design of the 3DTIPs.

### 2.7 Advancing 3DTIPs with FIB Sharpening and CNT Integration

After investigating high speed limits of the 3DTIPs, our next aim was to further improve the imaging resolution by advancing the 3DTIPs. To accomplish this, we reduced the tip radius of the 3DTIPs by deploying two independent post‐processing procedures, namely FIB etching and CNT integration (see Experimental Section).

**Figure 6:**
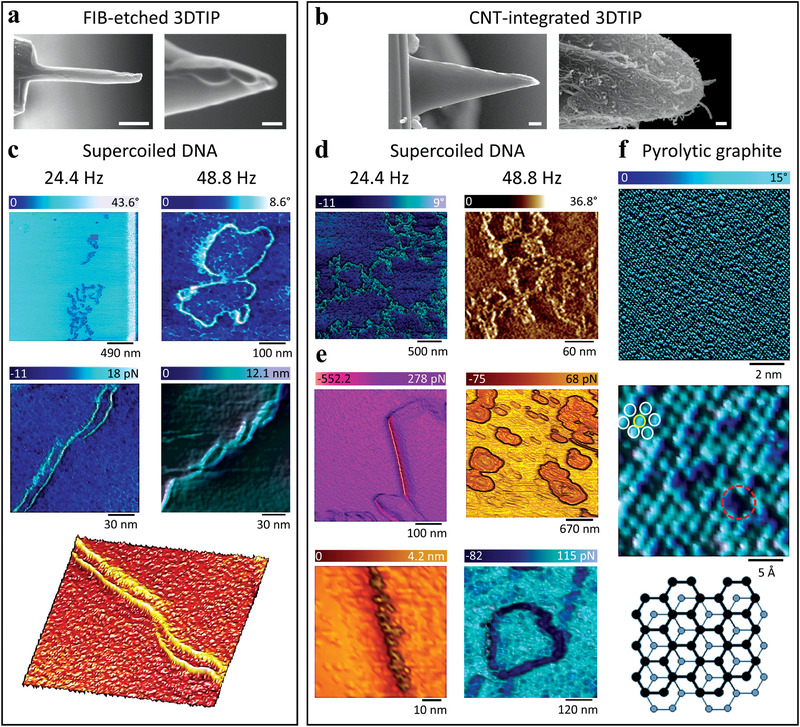
High resolution imaging performance of FIB‐etched and CNT‐integrated 3DTIPs. a,b) SEM images of the FIB‐etched and CNT‐integrated 3DTIPs used in the experiments. Scale bars: (a) 2 µm and 100 nm and (b) 4 µm and 200 nm (left to right). c) AFM phase (top two), adhesion (middle left and bottom), and height sensor (middle right) images obtained at different scales and scan rates with FIB‐etched 3DTIP in liquid using dynamic (top two) and PeakForce (middle two and bottom) modes. The supercoiled DNA (top right) and its plasmid rings (middle right) are revealed, with apparent thickness of ≈4.5 nm. The 3D representation of the plasmid in the adhesion image is also shown in bottom. d) AFM phase images obtained at different scales and scan rates with the CNT‐integrated 3DTIP in liquid using dynamic mode. e) AFM peak force error (top two), height sensor (bottom left), and adhesion (bottom right) images obtained at different scales with CNT‐integrated 3DTIP using PeakForce mode. When compared to performance of FIB‐etched 3DTIP in height sensor image in (c), the apparent plasmid DNA thickness of ≈6.2 nm is revealed in height sensor image in (e). f) The true atomic resolution (with atomic distance of ≈0.3 nm) is achieved on HOPG with CNT‐integrated 3DTIPs in liquid using dynamic mode (scan rate = 24.4 Hz). The hexagonal structure (white circles) and point defects (i.e., voids, the dashed red circle) are resolved. The schematics (bottom) show the highly ordered structure of HOPG. The drawing is not to scale.

In the first approach, FIB was deployed to etch (mill) sections of the tip end to sharpen it and achieve smaller tip radius, thus increasing the aspect ratio of the 3DTIPs (Figure 1c). For example, using FIB etching, we reduced the tip radius of a standard 3DTIP from 150 to 30 nm, while increasing its aspect ratio from 2 to 15 (**Figure** **6**a). During etching, Ga^+^ ions induce massive heat in the tips due to the low thermal conductivity of SU8, and cause amorphization of their surface as a result of ion implantation.^[^ ^40^, ^41^ ^]^ As a result, the microstructure and composition of the tip are altered, and thus its mechanical properties.^[^ ^41^ ^]^ However, such alterations are not considered to substantially affect the quality of AFM images as long as the cantilever is not “damaged” during the FIB etching in relation to its deflection characteristic. Nevertheless, detailed mechanical characterization of 3DTIPs’ cantilever and tip end under various milling optimizations, such as lowering the temperature effect or limiting the amount of implanted Ga^+^ ions as well as the implantation depth, is left for future work.

In the second approach, the 3DTIPs were carefully dipped into a pile of randomly oriented CNTs and the affixation of CNTs was achieved by UV curing (Figure 1d). This way, the tip radius of 3DTIPs was further reduced down to ≈6.5 nm (Figure 6b). Thus, the integration of CNTs to 3DTIPs provides excellent route for reducing the tip radius. However, their integration is not necessarily required as basis for production of HAR 3DTIPs. For example, in a separate approach, the tip radius and aspect ratio of 3DTIPs can be improved by deploying FIB induced deposition of tungsten onto tipless 3DTIP cantilever followed by FIB etching.^[^ ^42^ ^]^ As a result, tips with 5 nm radius and 1:30 aspect ratio can be produced.^[^ ^42^ ^]^ Although, this approach would add additional step to the FIB procedure, fabricating higher aspect ratio tips on SU8‐based cantilevers would come as an added advantage for high resolution, high speed AFM imaging.

Following the modifications, we first tested the FIB‐etched 3DTIPs for high resolution imaging of plasmid DNA in liquid using dynamic mode. The phase images in Figure 6c were obtained at relatively high scan rates (24.4 and 48.8 Hz), which resulted in apparent DNA thickness of 4.5 ± 1.2 nm (*n =* 5). Same images also revealed the distinct morphology of a supercoiled DNA, which in comparison to HAR 3DTIPs reinforced improved imaging resolution using FIB‐etched 3DTIPs. As such, from quantitative perspective, the ratio of apparent thicknesses of the DNA produced by HAR and FIB‐etched 3DTIPs to its true thickness (≈2 nm^[^ ^36^ ^]^), that is the ratio of “exaggeration,” read ≈15 for HAR 3DTIP and 2.2 for FIB‐etched 3DTIP (with DNA apparent thicknesses of ≈30 and 4.5 nm, respectively). Thus, sharpening further the HAR 3DTIPs with FIB etching evidently improved the imaging resolution, making the FIB‐etched 3DTIPs suitable for more accurate bio‐molecular measurements. Imaging of various other HAR structures, on the other hand, is left for future work.

AFM has long been perceived as the most suitable tool to dynamically investigate DNA molecules and their interaction with protein, owing it to its high resolution imaging and ability to characterize DNA in its physiologically relevant environment. However, AFM investigations of DNA with conventional silicon tips have been plagued with a myriad of challenges, some of which are directly attributed to the nature of silicon.^[^ ^43^, ^44^ ^]^ For example, on one hand, when DNA is immobilized on a substrate (such as mica), strong fixation forces could result in a suppression of DNA's physiological activity. While on the other hand, if the fixation forces are too weak, the silicon tip could easily dislocate the DNA.^[^ ^45^ ^]^ Therefore, during imaging soft biological samples such as DNA, the forces of interaction between the tip and the sample should be kept small (<100 pN). This fact additionally necessitates the need for the softer 3DTIPs with lower Hamaker constant in investigations that require lowering the interaction forces.

Clearly, compared to HAR 3DTIPs (Figure 4d,e), the enhancement in the imaging resolution of DNA with FIB‐etched 3DTIPs was obvious. For further comparison, however, we next utilized the CNT‐integrated 3DTIPs in imaging the DNA with scan rates similar to FIB‐etched 3DTIPs and using dynamic (Figure 6d) and PeakForce (Figure 6e) modes. In both modes, resulting DNA images produced similar resolution to the FIB‐etched 3DTIPs, where the apparent DNA thickness was measured to be 6.2 ± 3.2 nm (*n =* 5), which corresponded to exaggeration ratio of ≈3.1.

Beyond providing high resolution to fine 3D nanostructures, AFM has also led to achieving apparent atomic resolution on conductors^[^ ^46^ ^]^ and insulators.^[^ ^47^, ^48^ ^]^ In these studies, AFM permitted the observation of the periodic lattice spacing of the samples, yet resolving structure defects, such as single defects and step edges, were difficult to obtain. It was generally assumed that the limitation preventing true atomic resolution was due to multiple atom‐based tip‐sample interactions.^[^ ^49^ ^]^ Therefore, with the CNT‐integrated 3DTIPs, we targeted the true atomic resolution by reducing the tip radius, which minimized the number of atoms interacting with the sample of interest.

Additionally, attempts to measure subatomic structures are generally limited by multiple tip‐related artifacts, such as the geometry and shape of the tip, its material‐related structural defects, and the presence of tip surface contamination. In this context, by integrating CNTs with the 3DTIPs, not only we reduced their tip radius, but also took advantage of physical properties of CNTs that are ideal for minimal tip‐related artifacts: namely, HAR, large Young's modulus of elasticity, and low surface energy.^[^ ^18^ ^]^ As a result, with the CNT‐integrated 3DTIPs we were able to reveal (with atomic distance of ≈0.3 nm) the lattice structure, dimension, and atomic packing density of HOPG^[^ ^50^ ^]^ in liquid using dynamic mode (Figure 6f).

Moreover, with CNT‐integrated 3DTIPs, we were also able to observe voids in the HOPG that resembled single point defects (Figure 6f). It is evident that the superior mechanical properties of the CNTs, as well as their minimal thermal fluctuation, reflect in a highly stable scanning performance. For example, in single‐setup imaging experiments, CNT‐integrated 3DTIPs proved stable performance with multiple scans at atomic resolution both in air and liquid. However, it should be bared in mind that when working in liquid, tip contamination or loss of far‐reaching CNTs could occur during either tip immersion or tip extraction. Therefore, to minimize these effects it is recommended to prepare fresh tips especially after using them in liquid.

We hypothesize that achieving the true atomic resolution with CNT‐integrated 3DTIPs, by which single point defects are also observed, is majorly attributed to the presence of fewer atoms or even a single far‐reaching atom at the tip end of the CNT, thus allowing to fully resolve atomically flat surfaces such as of HOPG. It is also noteworthy to mention that the force contribution of SU8 side of the 3DTIP end is completely screened (i.e., diminished). Indeed, factors such as SU8's extremely low Hamaker constant, the liquid environment in which the 3DTIP is immersed, and the relatively large separation distance from the sample surface (≈200 to 300 nm) result in overall tip‐sample interaction that is fully dominated by the tip end of the CNT and the atomically flat surface. Thus, with the CNT‐integrated 3DTIPs common factors such as thermal noise and jump‐to‐contact that limit the amplitude ranges are all overcome, making true atomic resolution achievable.

## 3 Discussion

**Figure 7:**
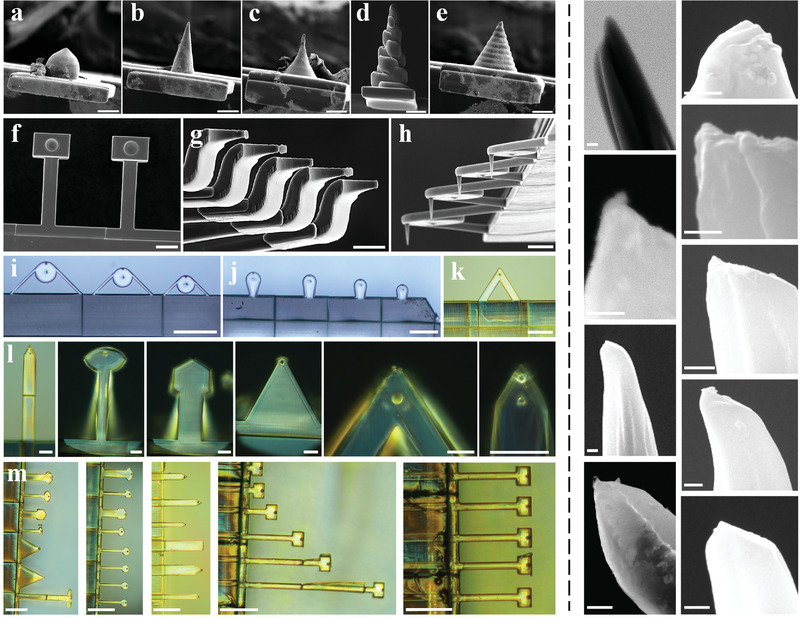
Multipurpose 3DTIPs with great flexibility in design and function. Left panel: a–h) SEM and i–m) optical images showing the variety of tip and cantilever shapes in the 3DTIPs that can be generated using 2PP. Depending on the design, the printing time of the 3DTIPs comprising mounting base and five cantilever‐mounted tips can be as low as 35 min to as high as 1.5 h. Clearly, with the proper tip and cantilever design, the AFM applications of 3DTIPs could range from probing forces to high‐resolution, high speed imaging. Scale bars: (a–e) 10 µm, (f–h) 40 µm, (i–k) 120 µm, (l) 30 µm, and (m) 120 µm. Right panel: Magnified SEM images showing the tip end of different HAR 3DTIPs. Scale bars: 100 nm.

In this work, we developed the technology for 3D generation of multipurpose AFM tips, and showed that 2PP can be effectively deployed for rapidly fabricating polymeric 3DTIPs in a variety of innovative designs and shapes (**Figure** **7**). These 3DTIPs are to inspire various AFM applications. For example, the 3DTIPs with beads (Figure 7a) could be utilized in probing viscoelastic properties of cells (Figure S8, Supporting Information). While with this work, the conical and HAR 3DTIPs in Figure 7b,c demonstrated excellent capabilities for high resolution, high speed AFM imaging.

Various other tip and cantilever shapes, as presented in Figure 7d‐m, emphasize the limitless design ideas one can lean toward producing multipurpose AFM tips. For example, by introducing a disk swing structure to the 3DTIP cantilever design such as shown in Figure 7i, two oscillation modes can be produced, one resulting from the cantilever arms and the other from the oscillatory disk. These 3DTIPs could then be used in bimodal AFM applications, where information about the sample depth could be extracted.^[^ ^51^ ^]^

Compared to silicon tips with similar design, the 3DTIPs outperform in terms of achieving high resolution images (even at high speeds) with minimal contamination and isotropic tip wear. As explained by the Hamaker approach, this attribute is due to significantly smaller and fast decaying interaction forces between the 3DTIP and the sample surface, especially when working in liquid environment. Large interaction forces are attributed to a large portion of the tip volume interacting with the sample. This results in coarser images and as well brings a higher probability of jump‐to‐contact. Moreover, for biological applications such as measurements performed on proteins or fast scans of DNA molecules, large interaction forces also produce irreversible sample deformations with corresponding loss of resolution. In contrary, small interaction forces result in higher resolution due to more localized tip‐sample interactions. Since AFM is a very sensitive tool with 0.1 nm deflection resolution and femtonewton force sensitivity, reduced interaction forces are preferred to achieve higher resolution and overall improved characterization. In the case of silicon tips, which possess high surface energy, large part of the tip surface interacts with sample, resulting in large forces of interaction due to high Hamaker constant of silicon. In the case of 3DTIPs, the forces of interaction are concentrated at the very end of the tip due to smaller effective tip radius and contribution of tip volume interactions, both of which result from the lower Hamaker constant of SU8. Thus, the produced AFM signal in the latter case is still well‐detectable.

Compared to HAR silicon tips, the smaller tip radius and higher aspect ratio of FIB‐etched 3DTIPs provide better stability at the cantilever level, which results in higher lateral and vertical resolutions, respectively. Thus, the FIB‐etched 3DTIPs are utile for all biological experiments that require soft tip‐sample interaction with minimal bio‐contamination on the tips. For example, the FIB‐etched 3DTIPs proved more versatile in accurately contouring the DNA plasmid while fast scanning it. This was due to involvement of minimal forces of interactions which allowed to image the DNA without scrubbing it, even though the sample was only physically adsorbed to the substrate surface. As a result, DNA imaging with the FIB‐etched 3DTIPs provided accurate estimation of DNA dimensions and shape, thus expanding their applicability in biological research. Noteworthy, for measurements involving imaging of magnetic samples and sensing soft magnetic matter, magnetic nanostructures can be further grown on FIB‐etched 3DTIPs using FIB induced deposition technique.^[^ ^52^ ^]^ This way, HAR ferromagnetic tips can be developed for better magnetic resolution and sensing. Production and characterization of such 3DTIPs, however, is left for future work.

The implementation of CNTs with silicon‐based AFM tips has been previously highlighted in terms of improved imaging resolution.^[^ ^18^ ^]^ This work, however, to our knowledge is the first to incorporate CNTs to SU8‐based AFM tips and investigate their functionality in terms of improved imaging resolution. Particularly, we show that with the CNT‐integrated 3DTIPs, the vertical and lateral imaging resolutions are significantly enhanced, especially when imaging highly ordered structures such as HOPG. Moreover, we also show that the CNT‐integrated 3DTIPs have the ability to robustly perform in air and liquid, and in common AFM modes. Our initial results proved that achieving the true atomic resolution with the CNT‐integrated 3DTIPs is possible when imaging atomically flat surfaces in ambient conditions. However, their further extensive characterization is still needed, which is left for future work.

Overall, in this work we showed that the 3D generation of multipurpose AFM tips is achievable, with great flexibility in design and function, using 2PP. Because the 3DTIP fabrication is in 3D and not 2D, 3DTIPs with various cantilever and tip geometries can be efficiently generated, allowing a better control over key AFM tip parameters, such as resonance frequency, quality factor, and spring constant. With our 3DTIPs, we further provided a direct insight on their performance for high resolution, high speed AFM imaging and compared their functionality with conventional silicon tips. We also highlighted multiple 3DTIP designs that cannot be obtained by the traditional 2D micromachining, but could be most useful when characterizing spatiotemporal resolution of various AFM measurements. Finally, we showed that FIB‐based sharpening and CNT‐mounting can be employed with innovative tip designs such as the ones shown in Figure 7. Therefore, the technology of 3DTIPs opens the way for innovative next‐generation AFM tips that are integrated and multifunctional.

## 4 Experimental Section

### 3DTIP Design, Fabrication, and Post‐Processing

The 3DTIPs were designed using a commercially available CAD software (SolidWorks). In the design, each 3DTIP comprised of a mounting base and five cantilever‐mounted tips. The cantilevers were designed 200 µm long, 30 µm wide at the mounting position and 60 µm wide at the tip position, and 2 to 10 µm thick. Key parameters (e.g., hatching and slicing distances, scanning speed, and laser power) were optimized to produce stable 3DTIP structure with minimum feature size (≈200 nm) and reduced surface roughness while maintaining fast printing time (<1.5 h). Worth to mention here that any other probe dimensions, number of cantilevers, and shapes of mounting bases are also possible.

The 3DTIP fabrication was performed in one step using a commercial 2PP system (Photonic Professional GT, Nanoscribe GmbH), where 30 × 30 mm^2^ cover slides (pre‐coated with indium‐tin oxide) were used as a substrate. During printing of 3DTIPs, a 780 nm‐femtosecond laser (near‐infrared light) was used to polymerize the UV‐sensitive photoresists (IP‐S resin, Nanoscribe GmbH), a 25× immersion lens with 0.8 numerical aperture (Zeiss LD LCI Plan‐Apochromat ImmCorr DIC M27 VIS‐IR) was used to focus the laser into resin, and ultrafast galvo‐mirrors were used to scan the laser focal point through the lens in *x* and *y* directions (GalvoScan mode) at 100 mm s^–1^ writing speed. This way, minimum feature size of 400 nm was obtained in the plane perpendicular to the laser beam. Depending on the system configuration, a high precision piezo stage could also be used (PiezoScan mode), and 63× immersion lens could be utilized for lateral resolution of <200 nm. However, printing time of the 3DTIPs in this case would be substantially longer. After printing, the 3DTIPs were soaked in propylene glycol methyl ether acetate bath, and in isopropanol to remove the non‐crosslinked polymer from the tip surface. Following, the top side of 3DTIP cantilevers was coated with a thin layer of gold. The generation of HAR 3DTIPs was achieved using same parameters. The 3DTIPs could be used right after coating them with gold. However, to enhance the tip dimensions and aspect ratio, 3DTIPs could also be further post‐processed either with FIB technology to produce FIB‐etched 3DTIPs, or with CNTs to produce CNT‐integrated 3DTIPs. In the former, 3DTIPs (coated with ≈10 nm gold layer using Cressington 108 Auto Sputter Coater) were transferred to a dual beam scanning electron microscope (ThermoFischer), where an FIB (Ga^+^ ion source, 30 keV beam voltage, 0.1 nA beam current) was used to systematically etch out their tip end. While in latter, multi‐walled CNTs with radius of 6.5 to 9 nm and length of 3 to 30 µm (Cheap Tubes Inc.) were integrated to the 3DTIPs by carefully dipping the tip end into a pile of CNTs. As such, the 3DTIP was mounted to XYZ linear motorized stage with built‐in controller (X‐LRM, Zaber Technologies), and a microscope glass slide with randomly positioned CNTs was placed on stage of an inverted microscope (Nikon Ti) equipped with 2× objective lens. The 3DTIP was then positioned on top of CNTs and approached until it was in contact with the CNTs. Upon 3DTIP retrieval, a cluster of CNTs remained temporarily pierced (attached) to the tip surface, after which the CNT‐integrated 3DTIP was transferred to UV curing chamber (Asiga Pico Flash) to permanently fix the CNTs.

### Finite Element Analysis

3D finite element method (COMSOL Multiphysics) was used to analyze 3D numerical models for the five cantilever‐mounted tip ensemble of the 3DTIPs. In the models, the five cantilever beams with varying thickness were imported as designed, and the boundary condition of the base was set as fixed. The cantilevers were modeled as linear elastic materials and the simulations were run for various cantilever dimensions by taking into consideration material properties of silicon (obtained from Materials Library, Institute of Making) and SU8 (with *ρ* = 1.02 g cm^–3^ and a Poisson ratio of 0.45 for cross‐linked IP‐L^[^ ^20^ ^]^). Each one of the five cantilevers was considered a simple harmonic oscillator, and the dynamics involved a simplification in discretizing the classical cantilever equation. Using a fourth‐order partial differential equation of motion, the flexural resonance frequencies, *f*_n_, were derived for homogeneous clamped‐free beams. Subsequently, its eigen frequencies were computed by a frequency domain solver using

(1) $$f_{n}=\frac{\lambda_{n}^{2}}{2\pi \sqrt{12}}\;\frac{H}{L^{2}}\sqrt{\frac{E}{\rho_{b}}}$$

where *λ*_n_ is the flexural mode (*λ*_1_ = 1.875), *H* and *L* are the cantilever thickness and length, and *E* and *ρ*_b_ are the Young's modulus of elasticity and density of the cantilever material, respectively.^[^ ^24^ ^]^

For cell stiffness measurements (Figure S8, Supporting Information), the cantilever beam was designed with a bead (5 µm diameter) at its end and the cell design resembled the one in its adhering state. The cantilever beam was then modeled as a linear elastic material both for silicon and SU8, the beads‐free end of the cantilever was fixed, while the cantilever system as whole was set as a moving boundary in the *y*‐axis with a 100 nm displacement step size. The whole cell was modeled as hyper‐elastic material with cytoplasmic and nucleic regions having elastic moduli 1 and 5 kPa, respectively. The boundary condition of the bottom surface of the cell was fixed. A contact point of coupling was set between the bead end point and the cell top surface point. Following, the static solver and contact mechanics were deployed to compute the cell deformation and the cantilever deflection at each indentation step.

### SEM Imaging

3DTIPs were coated with ≈10 nm gold layer using Cressington 108 Auto Sputter Coater. Following, SEM imaging was carried at 5 keV accelerating voltage using Cambridge S360 scanning electron microscope (Leica). The images were acquired digitally using UltraScan software (UltraScan Project).

### AFM Sample Preparation

For characterization of the 3DTIPs, PS spheres (Sigma Aldrich) with sizes of 30, 50, 80, 100, and 200 nm were deposited on a freshly cleaned glass slides and left to dry for an hour before imaging. For plasmid DNA imaging, a mammalian expression vector for expression of GFP (CMV promoter pCMV‐GFP, Addgene Inc.) was first diluted in 1 mL of phosphate buffered silane (PBS). The diluted solution was then deposited on a freshly cleaved mica, which was later glued on a glass slide. In order to minimize the evaporation, the glass slide was placed in humidifying chamber and incubated at 37 °C for 20 min. The unbound molecules were then gently washed with PBS and mica was covered with a thin layer of PBS for further AFM imaging.

### Glass Substrate Cleaning and Activation

AFM imaging was conducted using commercially available Dimension Icon (Bruker). Prior to imaging samples, the 3DTIPs were first thermally analyzed, from which information about their resonance frequency, quality factor, deflection sensitivity, and spring constant was obtained. Following, the deflection sensitivity and the spring constant were further calibrated by performing several force ramps on the glass substrate. AFM imaging was then performed in air or liquid using contact, dynamic, or PeakForce modes.

In all imaging, the tip‐sample interaction was detected through the deflection of the cantilever. In contact mode, the deflection was measured and compared to the desired constant deflection (set at 10 nm) by the feedback. In dynamic mode, the cantilever was excited and set to resonate at its damped natural frequency (i.e., its frequency of free resonance) in air or liquid. The drive amplitude (*A*) and amplitude setpoint (adjustable to 90% of *A*) were selected based on the nature of experimentation. For instance, for high speed imaging experiments, a minimum amplitude of 3 nm was used, while this value was reduced to 0.5 nm in case of atomic resolution imaging. The integral and proportional gains were also adjusted based on the scanning conditions. In PeakForce mode, on the other hand, the imaging was performed after calibration of the sync distance (at 29.21%) and theamplitude sensitivity (at 727.93 nm V^–1^) for each desired frequency. Following, the frequency (2 kHz) lower than that of the cantilever (62 kHz) was chosen, while the peak force amplitude and setpoint varied based on the sample conditions. For instance, when imaging DNA, the peak force setpoint varied between 10 and 50 pN.

### AFM Force Measurements

AFM force measurements were conducted using 3DTIP with ≈10 µm size bead and results were compared to conventional silicon tip with ≈6 µm size bead (CP‐PNP‐SiO, NanoAndMore). The spring constant of the 3DTIP cantilever was measured to be 0.04 N m^–1^ and that of silicon to be 0.08 N m^–1^. Prostate cancer cell line LNCaP, immobilized on a glass substrate through antibody–antigen interactions, was then probed for their viscoelastic properties in liquid using PeakForce mode, where 64 force curves were acquired while mapping 1 µm^2^ cell surface area with approach and retraction velocities set at ≈4 µm s^–1^. From acquired force curves, height, adhesion, stiffness, and modulus maps were then generated as described in the previous study.^[^ ^53^, ^54^ ^]^

### Data Analysis

Values were reported as the mean ± standard deviation (SD) from at least three repeats per experiment. AFM data were analyzed using Nanoscope Analysis software (Bruker). Plots and data fitting were generated using OriginPro data analysis and graphing software (OriginLab, USA). Statistical analysis was performed with OriginPro software using two sample *t*‐test analysis to evaluate FWHM differences between 3DTIPs and silicon tips. A *p*‐value of <0.05 was considered statistically significant.

## Conflict of Interest

The authors declare no conflict of interest.

## Supporting information

Supporting Information

## Data Availability

The data that support the findings of this study are available from the corresponding author upon reasonable request.
