# Supplementary material for: 3D Generation of Multipurpose Atomic Force Microscopy Tips
Source: Adv Sci (Weinh). 2022 Jul 19;9(27):2201489. doi: 10.1002/advs.202201489 (PMC9507387; doi:10.1002/advs.202201489)
Supplement: Supplementary file 1 — Supporting Information [file ADVS-9-2201489-s001.pdf]

ADVANCED SCIENCE
Open Access

Supporting Information

for *Adv. Sci.*, DOI 10.1002/advs.202201489

3D Generation of Multipurpose Atomic Force Microscopy Tips

*Ayoub Glia, Muhammedin Deliorman and Mohammad A. Qasaimeh**

© 2022 Wiley-VCH GmbH

Supplementary Information

3D Generation of Multipurpose Atomic Force Microscopy Tips

*Ayoub Glia, Muhammedin Deliorman, and Mohammad A. Qasaimeh**

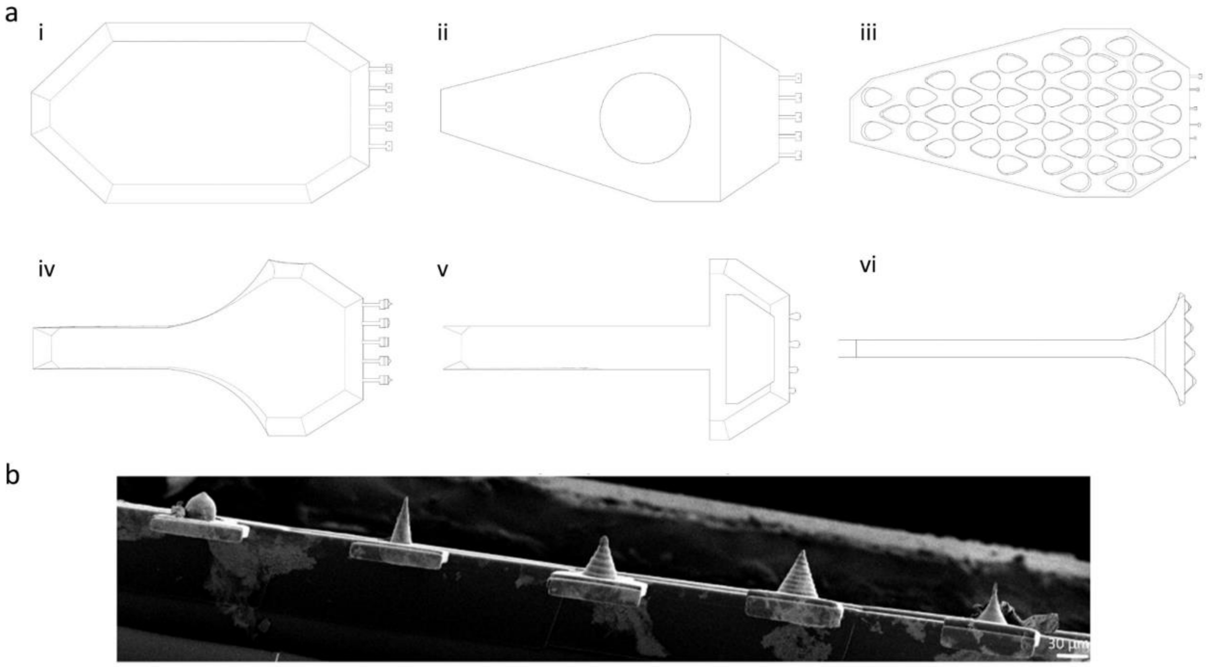

Figure S1. 3DTIP tip designs. (a) Different 3DTIP designs comprising mounting base and array of cantilever-mounted tips. i: Standard design (print time = 1.5 h). ii: Reduced design with ring cavity (print time = 1 h). iii: Eggshell reinforced design (print time = 1 h). iv: Arc design (print time = 45 min). v: Arrow design (print time = 30 min). vi: Miniaturized arc design (print time = 15 min). (b) SEM image shows a 3DTIP comprising 5 cantilever-mounted tips with different geometries.

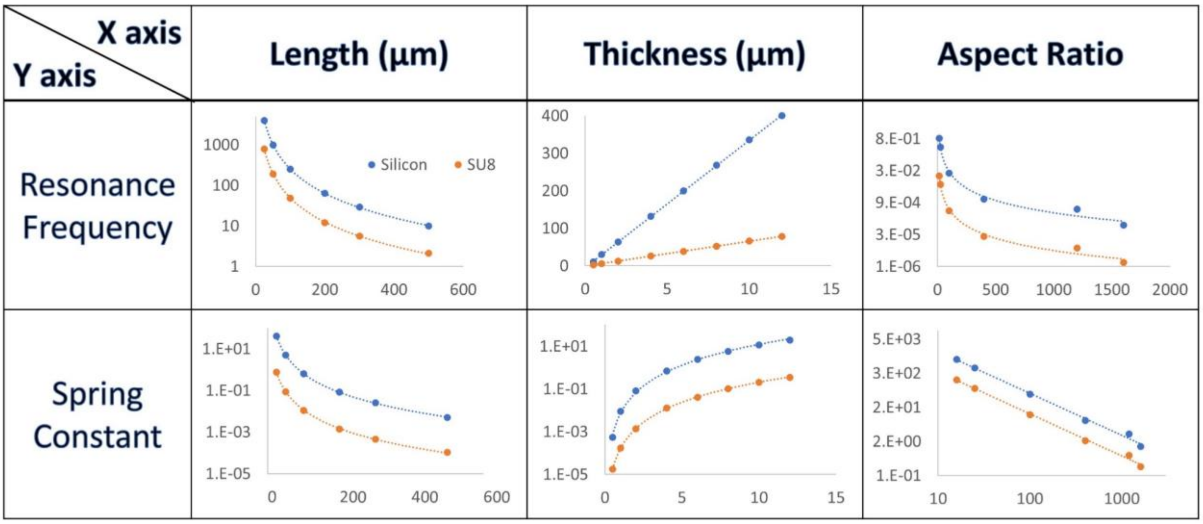

Figure S2. 3DTIP simulations. Simulation results comparing silicon-based (blue) and SU8-based (orange) AFM cantilevers in terms of the effect of the length, thickness, and aspect ratio of the cantilever on its resonance frequency and spring constant.

Table S1. Thickness, *H*, and length, *L*, values of 3 different cantilever designs, Type A, Type B, and Type C, used in investigating cantilevers' Q factor in air.

Type A		Type B		Type C	
H, μm	*L*, μm	*H*, μm	*L*, μm	*H*, μm	*L*, μm
3	125	7.5	127	4	150
3	100	5	105	4	125
3	75	3	80	4	100
3	50	2	66	4	75

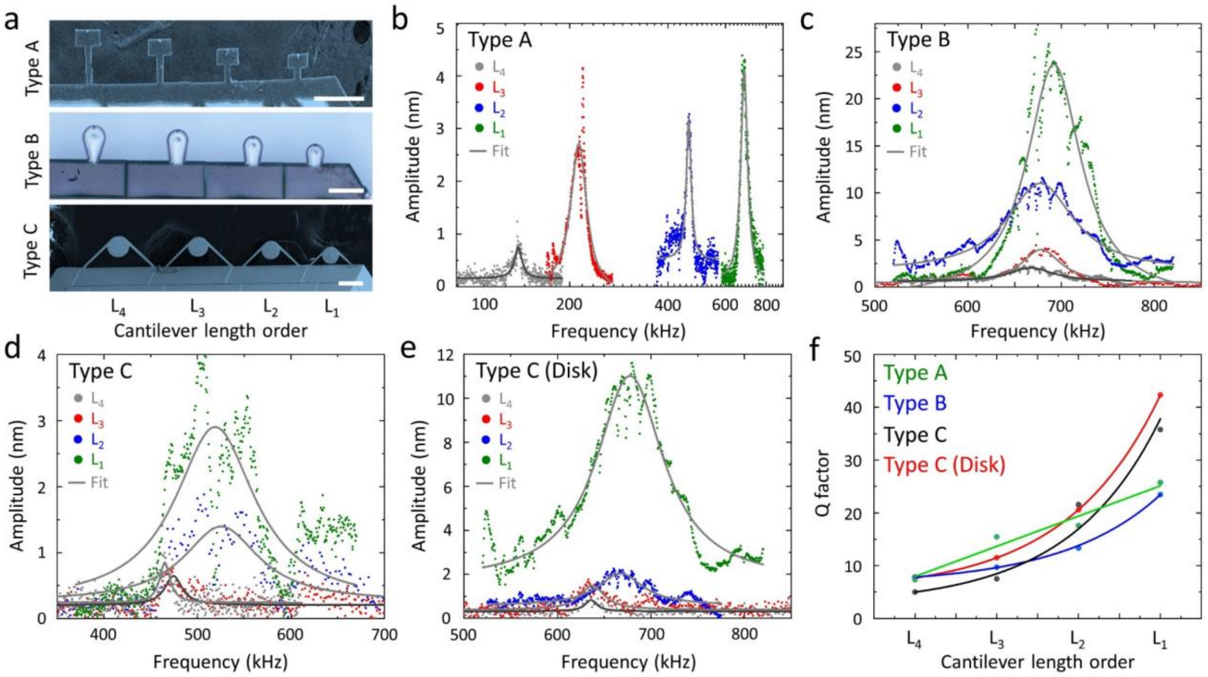

Figure S3. Parametric analysis of the Q factor for various 3DTIP cantilever designs in air. (a) Microscopy images show the Type A, Type B, and Type C cantilever designs used in the Q factor analysis. In Type A cantilevers (top panel), *H* was kept constant and *L* was linearly reduced; in Type B cantilevers (middle panel), *H* and *L* were both reduced while maintaining (H/L^2) ratio constant (constant frequency); and in Type C cantilevers (bottom panel), *H* was kept constant and *L* was linearly reduced. The design of Type C cantilevers comprise two legs and a disk resulting in a bi-modal fluctuation: one of the disk and one of the whole cantilever. Values of *H* and *L* are given in Table S1. L_4 to L_1 (cantilever length order) denotes the decrease in length from left to right. Scale bars are 120 μm. (b - e) Amplitude versus resonance frequency graphs show the experimental data (solid dots) for L_4 (grey), L_3 (red), L_2 (blue), and L_1 (green) of Type A, Type B, and Type C cantilevers, and the corresponding Lorentzian fit (solid grey lines) used in (f) estimating the Q factor values.

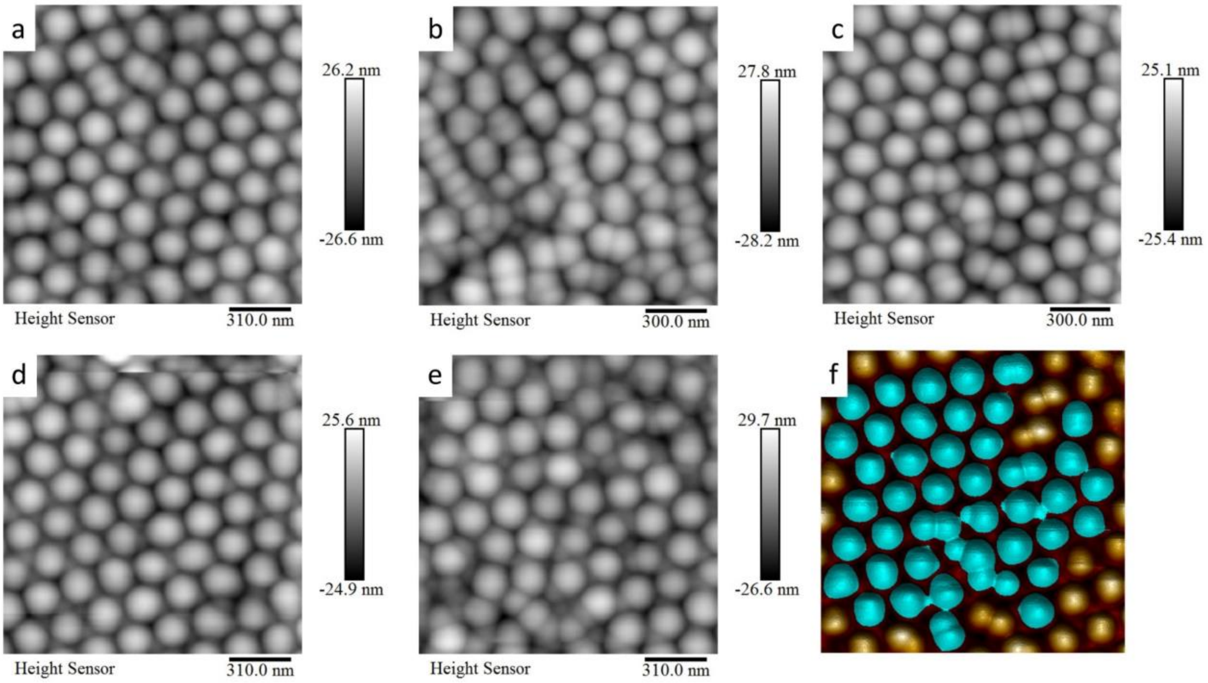

Figure S4. 3DTIP image reproducibility. (a-e) AFM height sensor images of 200 nm PS spheres obtained with a standard 3DTIP at different regions reveal consistent image reproducibility. (b) In AFM height sensor images, feature recognition is used to recognize the PS sphere shape and estimate its diameter.

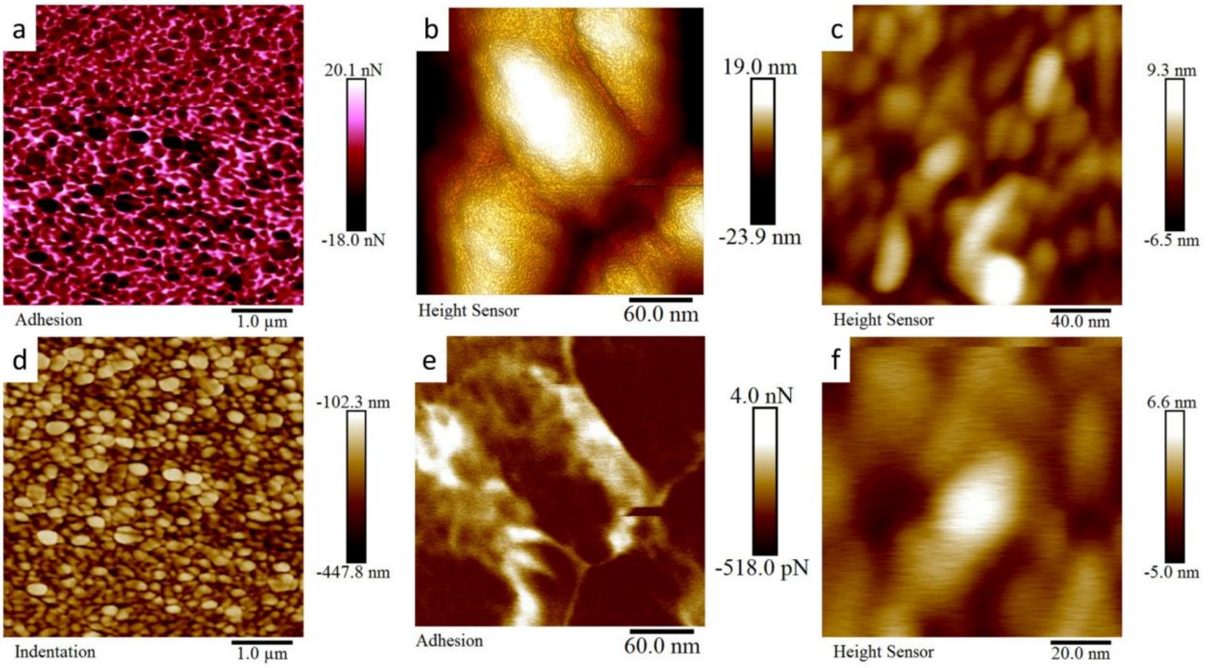

Figure S5. 3DTIP image quality of inorganic materials. (a-c) AFM adhesion and height sensor and (d-f) AFM indentation, adhesion, and height sensor images of the gold grains showing the image quality 3DTIPs can achieve for inorganic materials. ~20 nm grains size revealed with a 48.8 Hz scan rate.

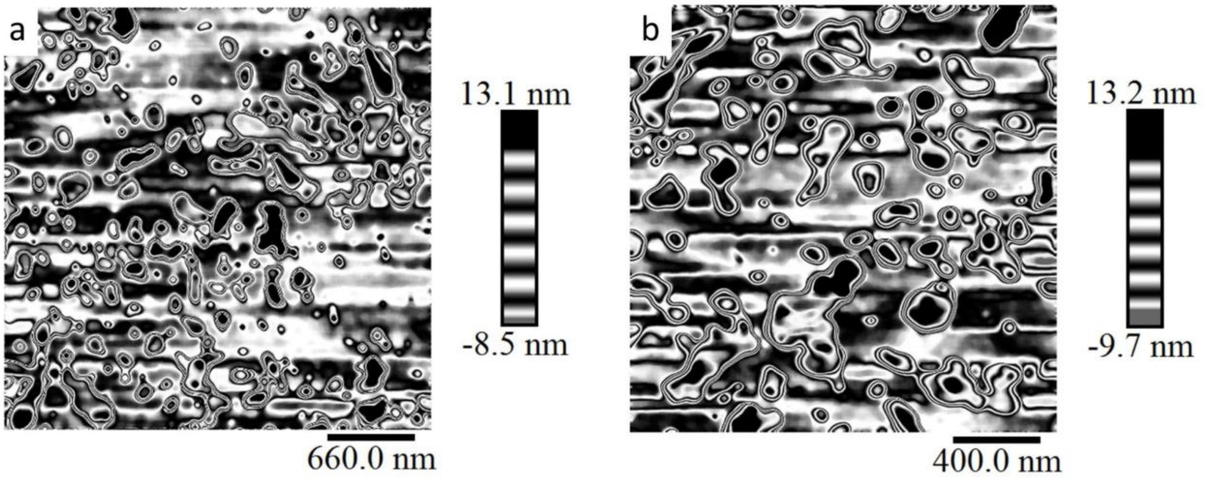

Figure S6. 3DTIP image quality of DNA. Comparison of the plasmid DNA imaging using (a) HAR 3DTIP and (b) HAR silicon tip. Clearly, HAR 3DTIPs (tip radius = 30 nm) reveal resolution comparable to HAR silicon tip (tip radius = 2 nm), with apparent DNA thickness of ~30 nm.

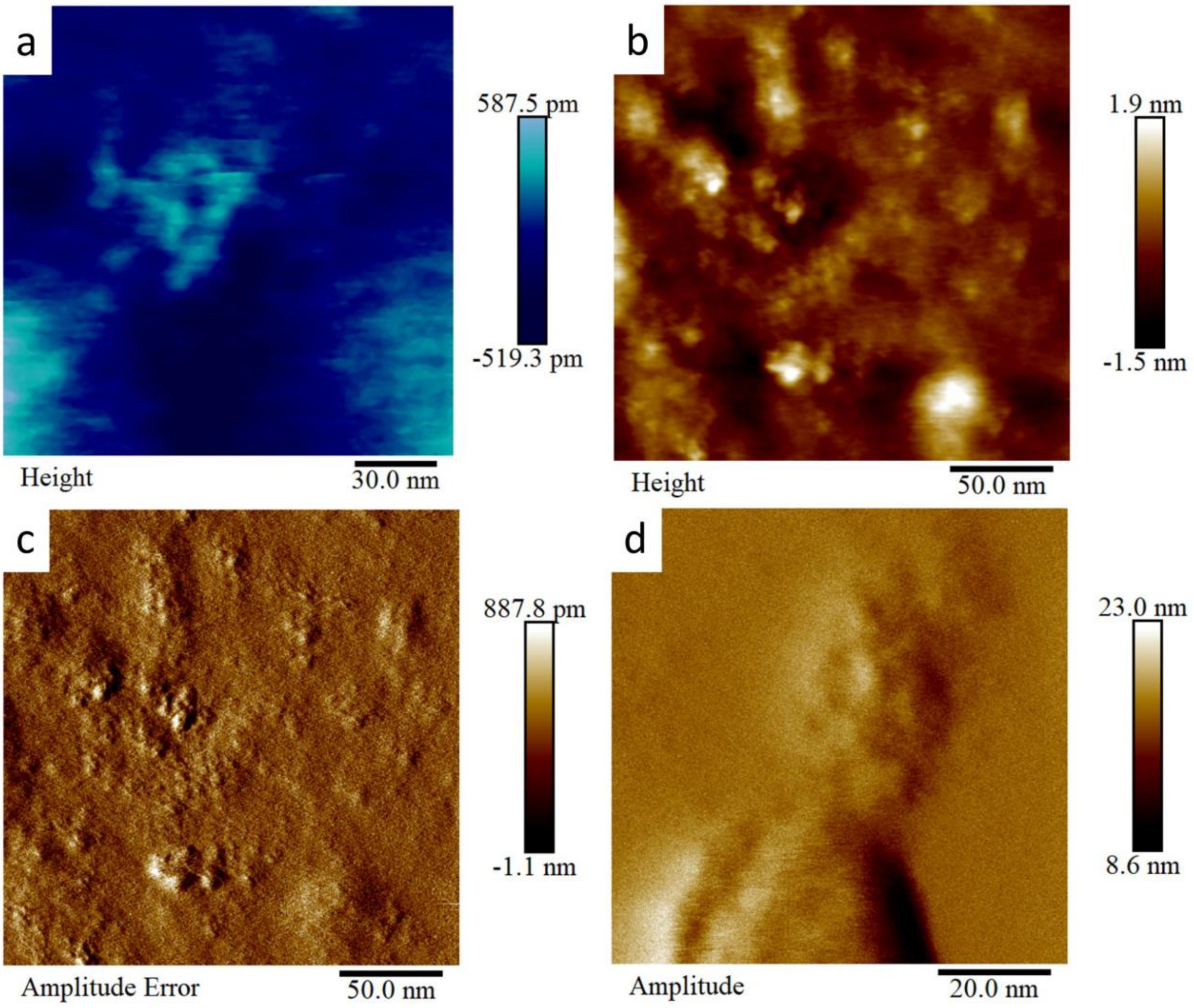

Figure S7. 3DTIP image quality of antibodies. (a-d) AFM height and amplitude images showing the epithelial cell adhesion molecule (EpCAM) antibodies at different scales. Their height (~2 to 6 nm) and commonly reported triangular shape is revealed in the images.

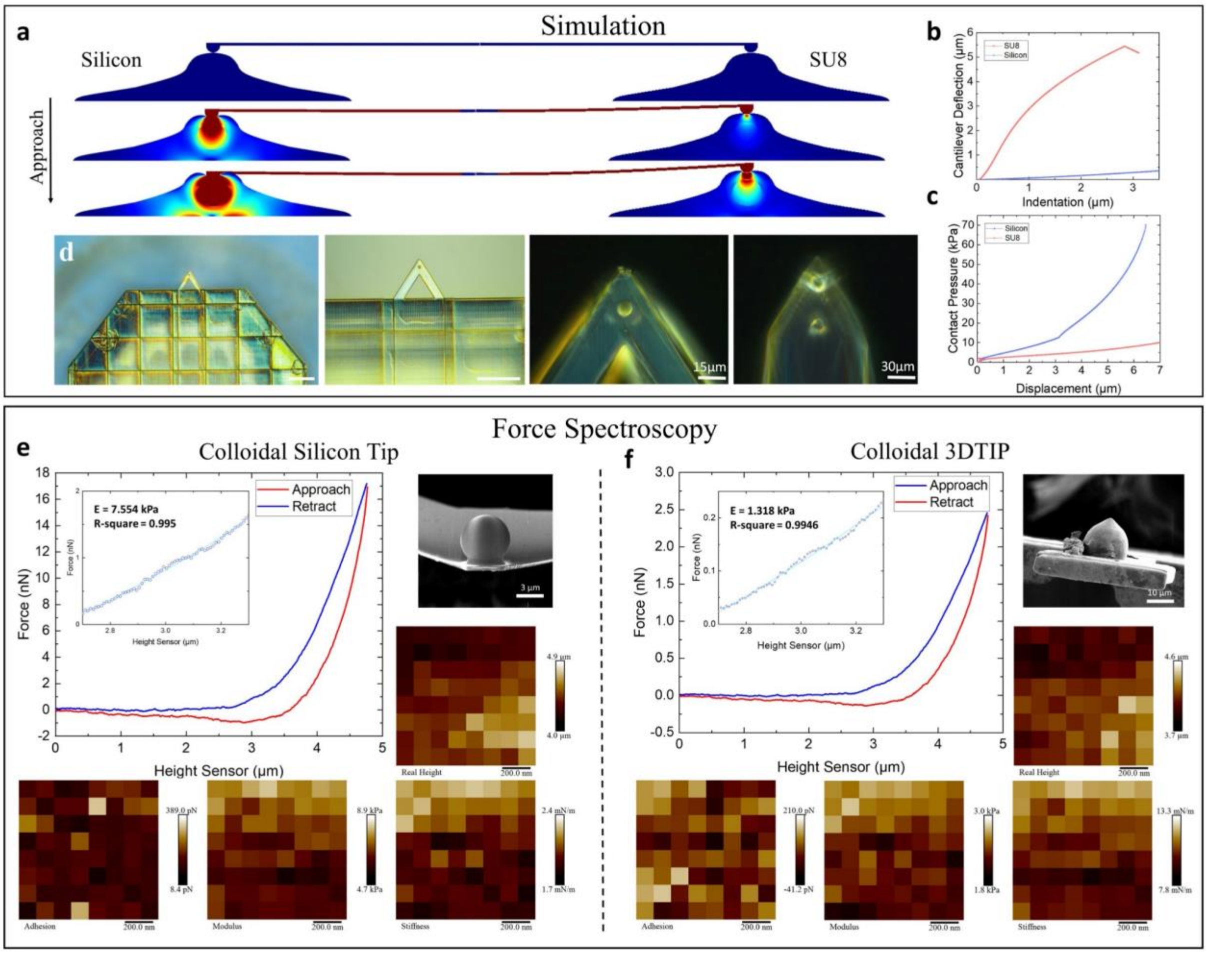

Figure S8. Comparison of cell stiffness measurements using silicon tip and 3DTIP by means of finite element and experimentation. (a) Qualitative representation of the simulation results showing a cell being deformed by the indentation using silicon-based and SU8-based tips. Clearly, silicon-based tip is causing more deformation to the cell despite its minimal deflection. (b) Cantilever deflection versus indentation graph (simulation results) for silicon-based (blue) and SU8-based (red). (c) Contact pressure versus displacement graph (simulation results) for SU8 (orange curve) and Si tip (blue curve). It is clear from simulation results in (b) and (c) that 3DTIPs are excellent candidate for probing cell viscoelastic properties. (d) Optical microscope images of a V-shaped and a beam-shaped 3DTIP with embedded bead at the cantilever end. Scale bars are 150 µm in the first two images. (e), (f) Proof of concept force measurements on prostate cancer LNCaP cells. In the experiment, a 3DTIP with embedded bead was benchmarked against the silicon tip with similar design (SEM images in the insets). Benchmarking was then performed by taking into consideration the height, adhesion, elasticity, and stiffness maps obtained from the upper left edge of the LNCaP cell. 64 force measurement were acquired by mapping 1 μm^2 cell surface area. Representative AFM force curve demonstrates the interaction of the tips, where the red and blue arrows represent the tip's approach to and retraction from the cell surface, respectively. Insets: Loading force versus surface indentation curves (open blue circles) show the data fit (solid blue lines) to Hertz model.
